# Unsymmetrical 1,1-Bisboryl Species: Valuable Building Blocks in Synthesis

**DOI:** 10.3390/molecules25040959

**Published:** 2020-02-20

**Authors:** K. Naresh Babu, Fedaa Massarwe, Reddy Rajasekhar Reddy, Nadim Eghbarieh, Manuella Jakob, Ahmad Masarwa

**Affiliations:** Institute of Chemistry, The Hebrew University of Jerusalem, Edmond J. Safra Campus, Jerusalem 9190401, Israel; nareshba.kaluvagadda@mail.huji.ac.il (K.N.B.); fedaa.massarwe@mail.huji.ac.il (F.M.); rajasekhar.reddy@mail.huji.ac.il (R.R.R.); nadim.eghbarieh@mail.huji.ac.il (N.E.); Ella.Jakob@mail.huji.ac.il (M.J.)

**Keywords:** 1,1-bis(boryl)alkanes and alkenes, bismetallated organic compounds, Suzuki–Miyaura cross-coupling, chemoselective transformations

## Abstract

Unsymmetrical 1,1-bis(boryl)alkanes and alkenes are organo-bismetallic equivalents, which are synthetically important because they allow for sequential selective transformations of C–B bonds. We reviewed the synthesis and chemical reactivity of 1,1-bis(boryl)alkanes and alkenes to provide information for the synthetic community. In the first part of this review, we disclose the synthesis and chemical reactivity of unsymmetrical 1,1-bisborylalkanes. In the second part, we describe the synthesis and chemical reactivity of unsymmetrical 1,1-bis(boryl)alkenes.

## 1. Introduction

**Scheme 1:**
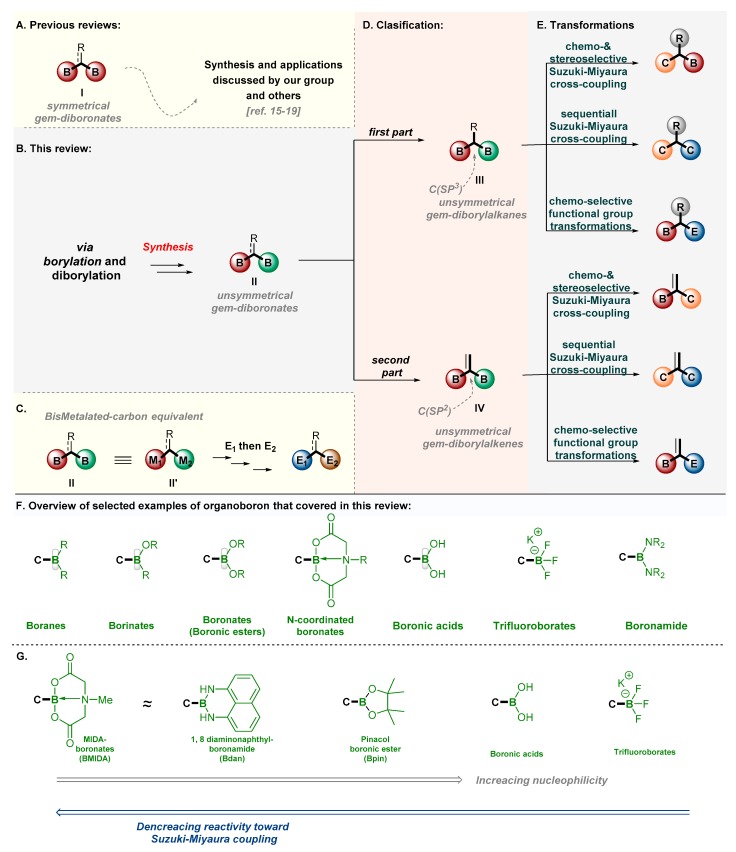
Overview of 1,1-bis(boron) species: their preparation and chemoselective transformations. (**A**) Previous works (**B**) This work (**C**) Bismetalated-carbon examples (**D**) Classification of gemdiboryl compounds (**E**) examples of selective transformations of gemdiboryl compounds (**F**) Overview of selected examples of organoboron that covered in this review (**G**) Reactivities scale of selected examples of organoborones bonds.

Over the last 70 years, organoboron compounds have dramatically changed the landscape of organic chemistry through a wide range of valuable and indispensable synthetic applications, e.g., cross-coupling chemistry, photochemistry, and alkylboration, which has led to new constructions of C–C and C–heteroatom bonds [1,2,3,4,5,6]. Additionally, boron functionality is dispersed in natural products and synthetic drugs. Natural products include the antibiotics aplasmomycin, boromycin, and tartolon B, in which boron functionality appears as a borate complex [7,8,9,10,11]. Drugs such as Tavaborole and Bortezomib also incorporate boron functionality [12,13,14]. In terms of these aspects, multiborylated compounds, e.g., **I**, are even more attractive due to their synthetic versatility and chemical stability, which allow for the selective synthesis of multifunctionalized molecules (Scheme 1) [15,16,17,18,19,20]. Recently, our group [16,17] and other research groups have mainly reviewed the new class of symmetrical 1,1-diboranes, **I** (bis-metallated reagents) (Scheme 1A,C), their preparation, and their application in organic synthesis for forming organoboranes and bifunctionalized products [15,16,17,18,19,20]. In contrast, the unsymmetrical 1,1-bis(boranes), **II**, have rarely been reviewed, despite their importance in the chemo- and stereoselective building of C–C and C–X bonds. These classes of compounds mainly include 1,1-bis(boron) (**III**) and alkenyldiboronates (**IV**) (Scheme 1B). These compounds offer two distinct boron substituents for ideal sites as well as stereoselective synthetic strategies to obtain stereo-controlled alkanes [16,17] and multisubstituted olefins, which are of major importance in organic synthesis [21,22,23,24,25,26]. Most importantly, 1,1-bis(boryl)alkenes have been utilized in the synthesis of the anticancer agent tamoxifen [27].

However, unsymmetrical 1,1-bis(boryl)alkanes (**III**) and -alkenes (**IV**) represent a unique class of boron compounds with a tunable reactivity for boron site-selective functionalization. This review will cover synthetic approaches for forming unsymmetrical 1,1-bis(boron) species as well as for deriving chemo- and stereoselective transformations for the synthesis of complex molecular structures. It is hoped that this review will provide useful knowledge for scientists seeking to discover new things about unsymmetrical 1,1-bis(boryl)alkanes and -alkenes in organic synthesis. The synthesis of these unsymmetrical 1,1-bisboryls includes mainly the hydroboration of alkynes and alkenes. Unsymmetrical 1,1-bis(boryl)alkanes and -alkenes are mainly utilized in chemo- and stereoselective (sequential) cross-coupling reactions and other chemoselective transformations. In order to simplify things for the readers, we classified this review article into two categories based on the hybridization of the carbon attached to the 1,1-bis(boron) functionalities:

(i) Unsymmetrical sp^3^-centered type-**III**; and
(ii) Unsymmetrical sp^2^-centered type-**IV**.

In both cases, we discuss the synthesis and utility of the unsymmetrical 1,1-bis(boron) species in organic chemistry.

Before going into the discussion, let us first introduce the characteristics of the C–boron bond in terms of reactivity, on the basis of their substitution pattern. It is well known that the organoboron groups (see Scheme 1F) [28] have different reactivities due to the steric and electronic properties of the Lewis acidic boron moiety, which can be easily tuned just by changing the substitution around the organoboron group, which allows for diverse reactivity as a stoichiometric reagent and as a catalyst. For example, boron reagents behave like nucleophilic component is a Suzuki–Miyaura (SM) cross-coupling reaction; therefore, their reactivity depends on their nucleophilicity. Nucleophilicity scale of organoborons (Scheme 1G) shows that organoboronic acids and organo-trifluoroborates have greater nucleophilicity than do MIDA-boronates (MIDA = *N*-methyliminodiacetic acid), (1,8-diaminonaphthyl boronamide, and boronic esters (as described in Scheme 1G) [28]; thus, MIDA-boronates have less reactivity toward the SM cross-coupling reaction [3]. Therefore, the two different organoboron groups in 1,1-bis(boron) species tend to show two different reactivities and perform in a chemoselective manner.

## 2. Unsymmetrical sp3-Centered 1,1-Bis(boryl) Compounds: Synthesis and Applications

**Scheme 2:**
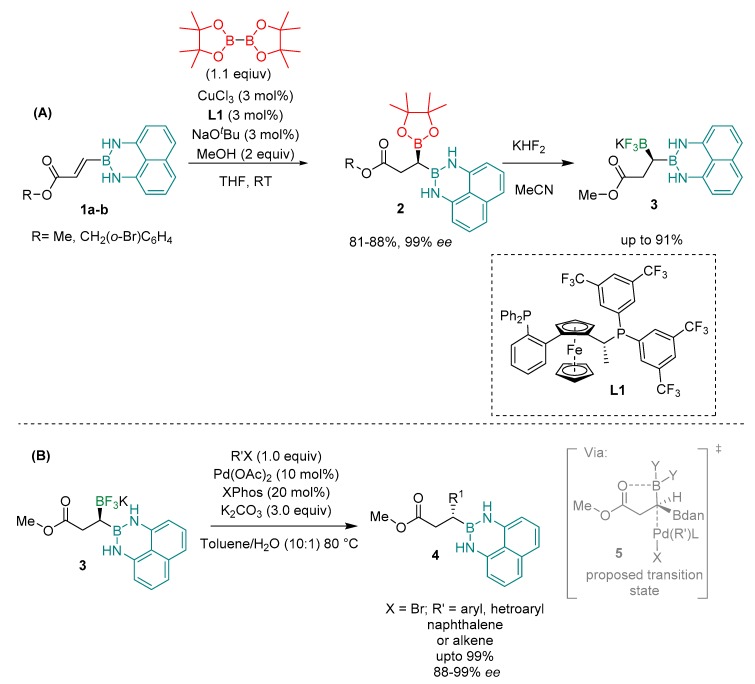
(**A**) Preparation of chiral 1,1-bis(boryl) carboxy esters via copper-catalyzed conjugate borylation. (**B**) Cross-coupling reaction of enantiomerically pure 1,1-bis(boryl) carboxy ester and the transition state for the transmetallation reaction.

In 2011, Hall and coworkers elegantly reported the first preparation of optically enriched unsymmetrical 1,1-bis(boryl) compounds (**2**) with excellent enantioselectivity via a copper-catalyzed asymmetric conjugate borylation of β-boronylacrylates (**1**) with pinacolatodiborane (Scheme 2A) [29]. The obtained enriched unsymmetrical 1,1-bis(boryl) compound (**2**) was then treated with KHF_2_, forming the corresponding trifluoroborate salt **3**. Of note, the Bpin (Bpin = B-pinacolato) group in **2** selectively underwent a trifluorination reaction over Bdan (Bdan = B-1,8-diaminonaphthalene) functionality, most likely due to the higher Lewis acidity available for activation of the p-orbital of the boron in Bpin compared to the lower Lewis acidity of the p-orbital of the boron in Bdan, which is aromatically busy (Scheme 2) [30]. An X-ray crystallographic analysis confirmed the conjugate borylation product of **2a** (R = Me); this provided us with a better understanding of the physical properties of these compounds.

In addition, the trifluoroborate salt **3** was stereo-specifically cross-coupled with aryl bromide in the presence of palladium catalyst and XPhos (2-dicyclohexylphosphino-2′,4′,6′-triisopropylbiphenyl) as a ligand: these salts formed the corresponding arylated product **4** in high yield and with good-to-excellent enantioselectivity (88–99% ee). The coordination of the carbonyl oxygen with the boron atom (see **5**) and the stabilization provided by the second boronyl unit in the α-B–Pd(II) complex are thought to facilitate the transmetallation process and the cross-coupling reaction. Interestingly, in this cross-coupling reaction, an inversion of the stereochemistry was observed. The rationale for this inversion was presented through a transmetallation reaction that took place via transition state **5**, which was responsible for inverting the stereochemistry (Scheme 2B) [29].

**Scheme 3:**
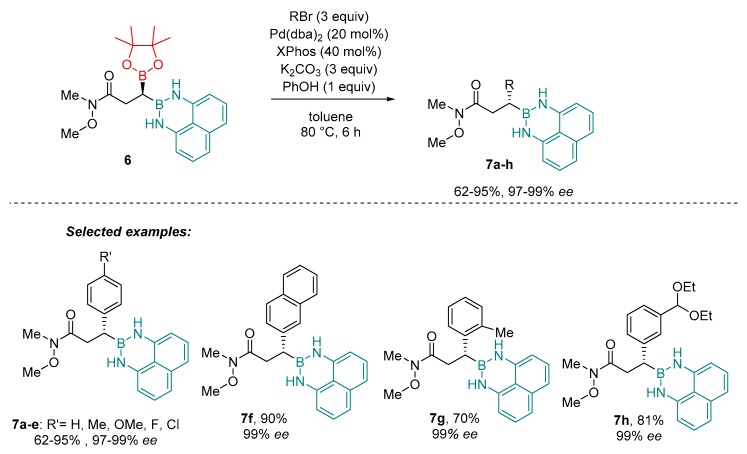
Cross-coupling reaction of chiral 1,1-bis(boryl)alkane with aryl bromides.

In 2015, this group expanded its investigations into palladium (Pd(dba)_2_) and the XPhos-catalyzed cross-coupling reactions of optically enriched unsymmetrical 1,1-bisboron compound (**6**), using aryl bromides in a chemo- and stereoselective manner for the synthesis of the benzylic secondary alkyl boronates **7a**–**h**, which had a stereochemistry inversion [31]. They also noted an important observation: by increasing the Lewis basicity of a carbonyl group from an ester to a Weinreb amide, the cross-coupling reaction was more feasible. The coordination of the carbonyl oxygen with the boron atom, together with the stability of the second boron unit in the α-B-Pd(II) intermediate, helped to overcome a difficulty at the transmetallation step. This reaction showed tolerance to a wide range of aryl coupling partners, with excellent chemoselectivity over the Bdan functionality (Scheme 3).

**Scheme 4:**
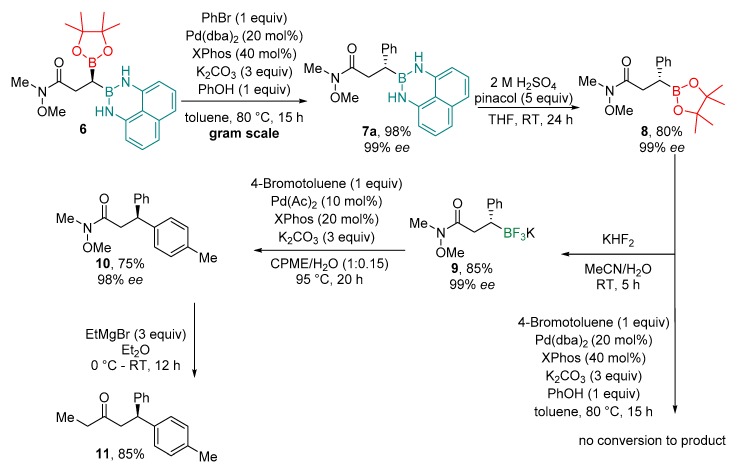
Synthetic transformations of chiral unsymmetrical 1,1-bis(borane) compounds.

These enantioenriched cross-coupled products were subjected to various synthetic transformations, as presented in Scheme 4. The Bdan motif was treated with acid, affording the hydrolyzed product, which further underwent esterification with pinacol, yielding compound **8**. Next, **8** was converted into its corresponding potassium trifluoroboronate salt, **9**; finally, it underwent a cross-coupling reaction with aryl bromide, affording compound **10** (with an inversion in its stereochemistry). The sequence of two cross-couplings, from **6** to **10**, was followed by double inversions of stereochemistry. Finally, amide **10** was transformed into ketone **11** upon a reaction with ethylmagnesium bromide (Scheme 4) [31].

**Scheme 5:**
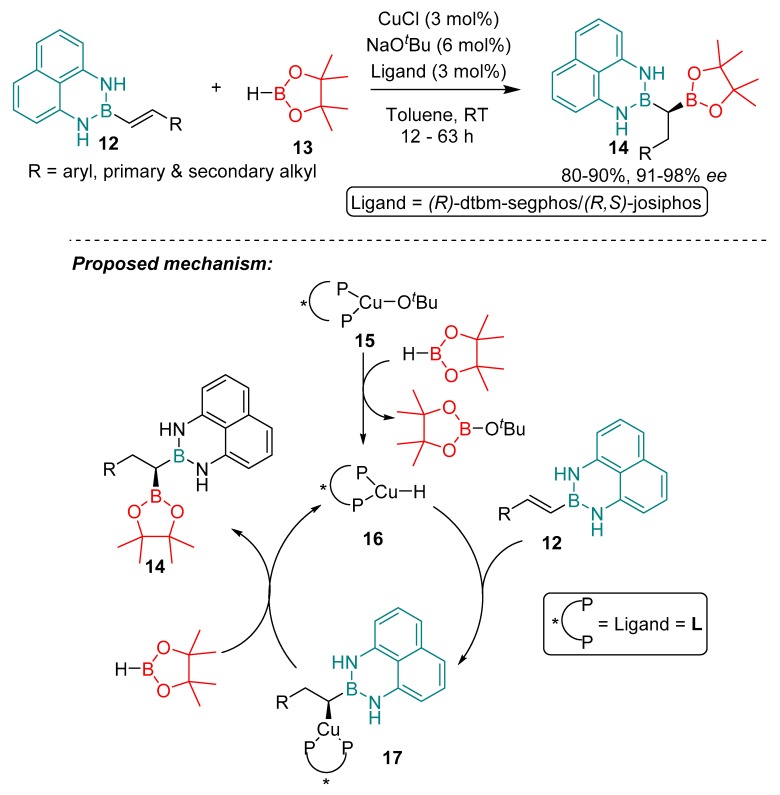
Copper-catalyzed enantioselective hydroboration of alkenes.

Yun and coworkers reported the use of copper-catalyzed hydroboration of borylalkenes to synthesize unsymmetrical 1,1-bis(boryl)alkanes in a high regioselective and enantioselective version [32]. Various alkene substitutions, such as aryl, primary, and secondary alkyls, were well tolerated in this reaction and provided excellent regioselectivity (Scheme 5). 

Initially, the reaction mechanism involved the reaction of copper *tert*-butoxide with pinacolatoborane, affording **L**Cu-H species **16**, which then underwent a regio- and enantioselective addition reaction into borylalkene **12,** yielding the chiral organocopper intermediate **17**. Next, this intermediate **17** underwent stereoretentive transmetallation with one more equivalents of pinacolatoborane, finally yielding the desired product **14** and regenerating the catalyst (**L**Cu-H) **16** for another catalytic cycle (Scheme 5).

**Scheme 6:**
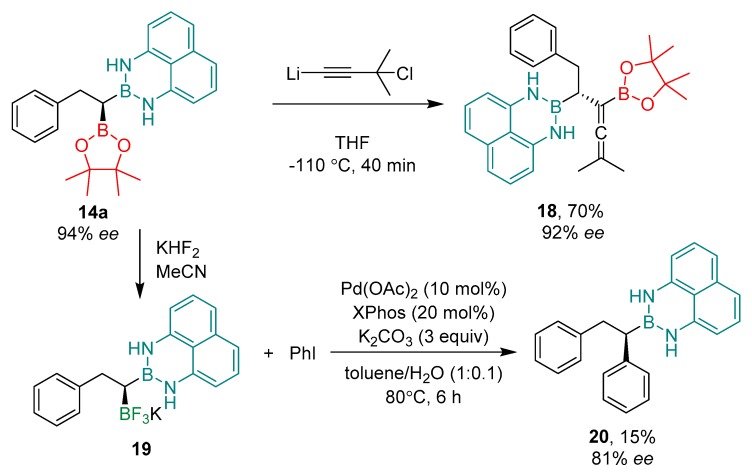
Synthetic transformations of chiral unsymmetrical 1,1-bis(boryl)alkanes.

These chiral unsymmetrical 1,1-bis(boryl)alkanes were then transformed into allenylboronate **18** in a homologation reaction with 3-chloro-3-methylbut-1-ynyllithium, with almost no erosion of its enantioselectivity. Similarly, the Suzuki–Miyaura cross-coupling (SMCC) of **19** afforded the corresponding arylated product **20** in a low yield and with little loss of its enantioselectivity (but with retention of its configuration) (Scheme 6) [32].

**Scheme 7:**
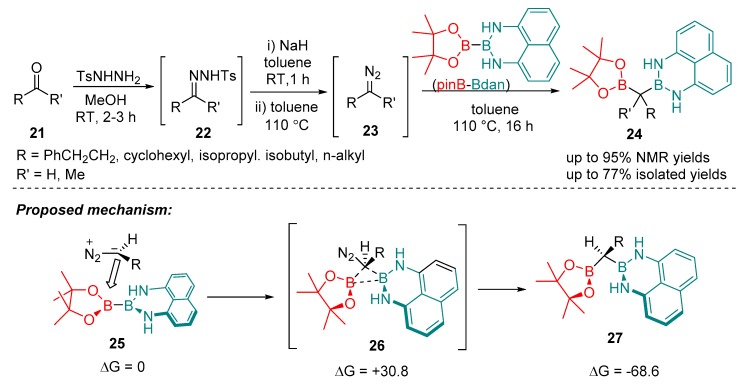
Metal-free insertion of diazo compounds into pinB–Bdan.

In 2015, Fernandez’s research group reported a transition metal-free approach for the synthesis of unsymmetrical 1,1-bis(boron) compounds from diazo compounds, **23**, which were obtained in situ from aldehydes as well as from cyclic and noncyclic ketones (**21**) via in situ-generated sodium salts of tosylhydrazones followed by treatment with sodium hydride (Scheme 7) [33]. This method also provides a wide range of unsymmetrical 1,1-bis(borane) compounds from aldehydes and ketones, with good isolated yields. However, it is strictly limited to aliphatic carbonyls.

In their work, they provided a rationalized mechanism for this method via transition state **26** (based on DFT-Density Functional Theory calculations). The reaction involved the heterolytic cleavage of the mixed diboron reagent **25** and the formation of geminal C–Bpin and C–Bdan bonds via a concerted–asynchronous mechanism, as shown in Scheme 7.

**Scheme 8:**
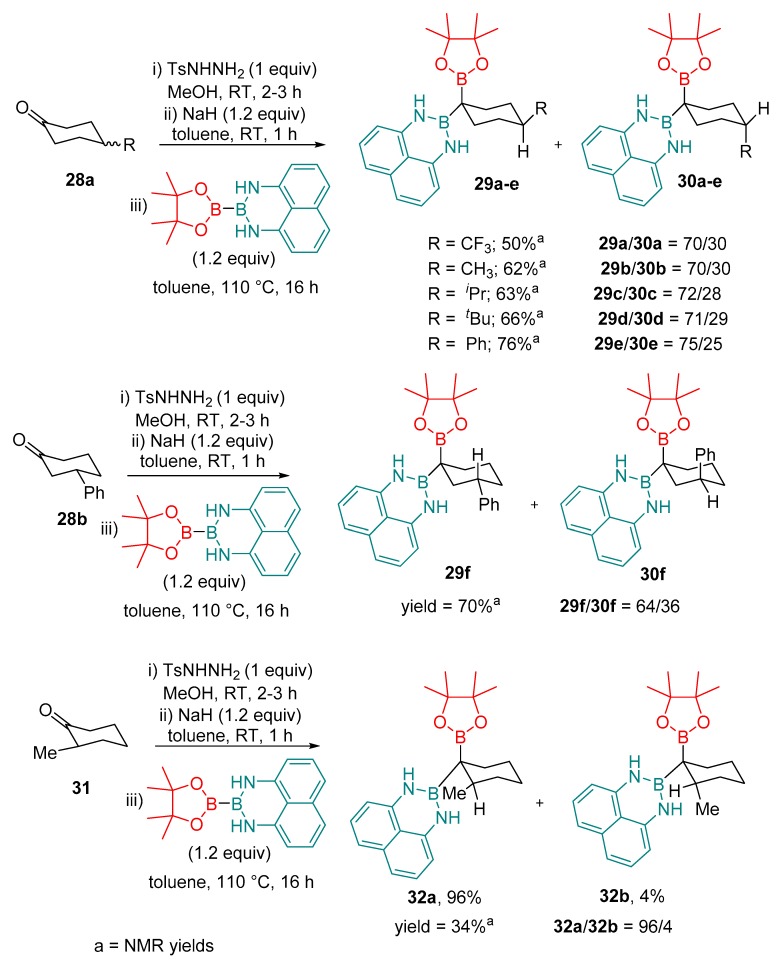
Diastereoselective unsymmetrical 1,1-diboration of substituted cyclohexanones.

To explore the diastereoselective 1,1-diboration of diazo compounds, Fernandez’s group substituted cyclohexanones for in situ-formed diazo-compounds and promoted insertion into the pinB–Bdan bond (Scheme 8) [33]. When they carried out a reaction with 4-substituted cyclohexanones (**28a**) under optimized reaction conditions, they observed reasonable diastereo-selectivity. An X-ray analysis of **29a** showed that the Bdan and CF_3_ groups were situated at the 1,4-diequitorial (trans) positions, as shown in Scheme 8. Under optimized conditions, the 3-Ph-cyclohexanone **28b** afforded the 1,3-diequitorial (cis)-substituted isomer as the major diastereomer **29f** (64%). Interestingly, under a similar reaction condition, the 2-Me-cyclohexanone **31** afforded excellent diastereo-selectivity (**32a**/**32b**), favoring the Bdan and Me groups with a 1,2-diequitorial (trans) configuration. This high diastereo-selectivity was expected because the 2-position substituent can directly influence the reaction center.

**Scheme 9:**
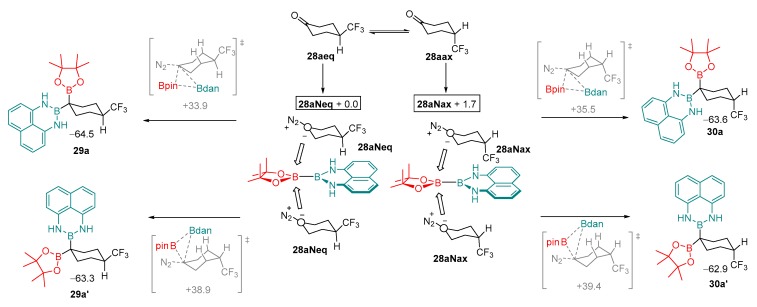
Proposed diastereo-isomeric pathways for the 1,1-bisboration of 4-CF_3_-cyclohexanone with pinB–Bdan and the relative Gibbs free energies in kcal/mol.

The origin of diastereo-selectivity was explained through DFT analysis by taking the example of the 4-(trifluoromethyl)cyclohexanone **28a**, as shown in Scheme 9. After the reaction of hydrazide and NaH with compound **28a**, Fernandez’s group considered in situ-generating two possible chair conformations (**28aNeq** and **28aNax**) that were different in terms of their CF_3_ substituent arrangement: the equatorial conformer (**28aNeq**) was 1.7 kcal/mol lower than the axial conformer (**28aNax**). Each confirmation could attack the pinB–Bdan substrate through its two diastereo-faces. When the conformer **28aNeq** attacked pinB–Bdan through its two diastereomeric faces, the observed computed free energy barriers for the Bdan equatorial and Bdan axial positions were 33.9 and 38.9 kcal/mol, respectively (Scheme 9, left side). Thus, the lowest energy path led to a major diastereomer with Bdan and a CF_3_ substituent in the 1,4-diequitorial (trans) of each **29a**. The high energy (~5.0 kcal/mol) in the Bdan axial approach was due to destabilized interactions between 1,3-diaxial and cyclohexane. Similarly, the conformer **28aNax** also exhibited a low energy barrier in the case of the Bdan equatorial approach, as opposed to the Bdan axial approach (Scheme 9, right side).

The energy difference between the two Bdan equatorial approaches was not too large in the two conformers (**28aNeq** and **28aNax**), with the expected non-negligible formation of conformer **30a** with an observed diastereomeric ratio of 70/30 for **29a**/**30a** [33].

**Scheme 10:**
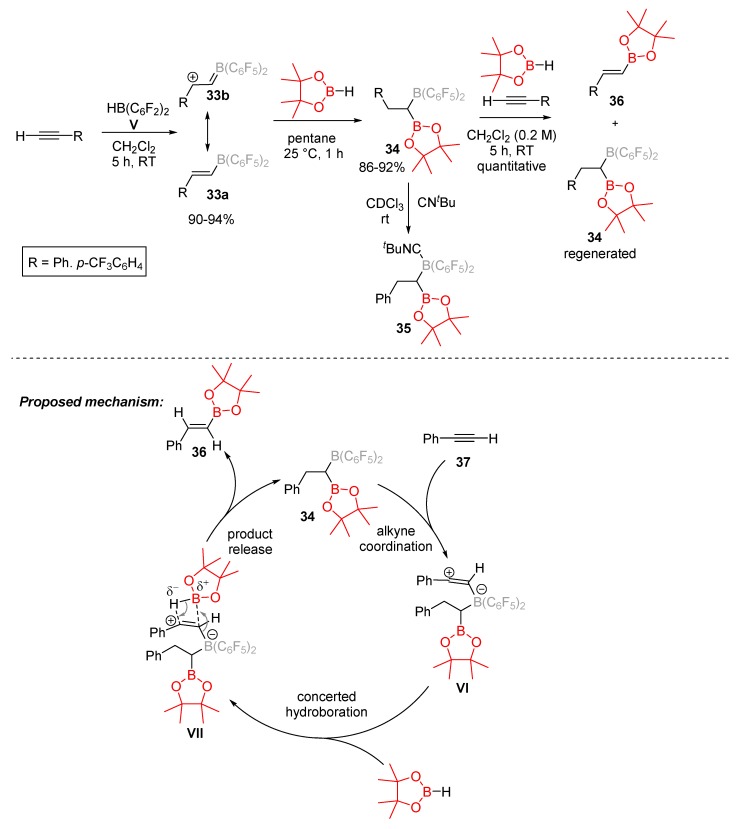
Unsymmetrical 1,1-bis(borane) catalyzing the hydroboration of alkyne.

Interestingly, 1,1-bis(boryl)alkanes can behave as catalysts, too. Piers’s borane **V** [HB(C_6_H_5_)_2_] precatalyzed the hydroboration of terminal and internal alkynes for the synthesis of *E*-alkenyl pinacol boronic ester **36**, and excellent selectivities were reported by Stephan and coworkers in 2016 [34]. In this hydroboration, they found that unsymmetrical 1,1-bis(borane) **34** catalyzed the reaction. An independent reaction of Piers borane **V** [HB(C_6_H_5_)_2_] with phenylacetylene afforded the corresponding *E*-alkenyl boronic ester **33a**, which, upon additional treatment with pinacol boranes, yielded the regioselective stereogenic unsymmetrical 1,1-bis(borane) **34** (Scheme 10). Actually, 1,1-bis(borane) **34** acted as an electrophilic catalyst for the hydroboration of alkyne into alkene in the presence of HBpin (pinacolborane). To support this, the electrophilic boron center of **34** was confirmed through an X-ray diffraction analysis of the *tert*-butylisonitrile adduct **35**.

The proposed mechanism for the unsymmetrical 1,1-bis(borane) **34** catalyzing the hydroboration of alkynes for the synthesis of *E*-alkenyl boronic ester **36** is shown in Scheme 10. In this mechanism, unsymmetrical 1,1-bisboranes **34** acts as a Lewis acid catalyst, which activates alkyne to form complex **VI**: then, HBpin reacts with complex **VI** in a concerted *syn*-1,2-hydroboration manner (complex **VII**) to afford the hydroborylated product **36** and regenerate the unsymmetrical 1,1-bis(borane) **34** [34].

**Scheme 11:**
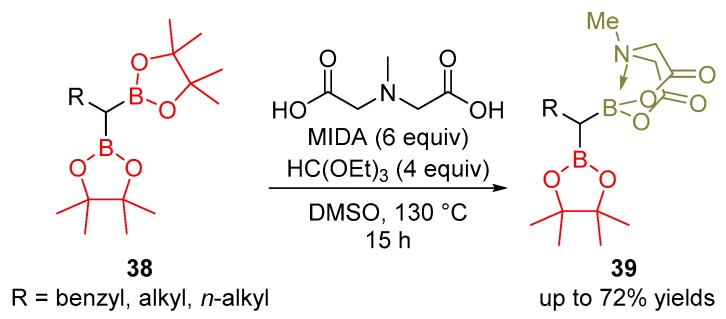
Synthesis of unsymmetrical 1,1-bis(borane) products.

In 2019, Sharma research group reported a novel and concise method for the synthesis of a wide range of MIDA (MIDA = *N*-methyliminodiacetic acid) acylboronates (**41**) via the chemoselective oxidation of 1,1-bisboranes (**39**) (Scheme 11) [35]. MIDA acylboronates are synthetically challenging; however, they can be utilized as powerful building blocks for bioorthogonal amide formation and protein ligation.

First, Sharma prepared unsymmetrical 1,1-bis(borane) products (**39**) from symmetrical diboranes (**38**) by heating MIDA and triethylorthoformate (Scheme 11). The mechanism underlying this selective desymmetrization is still unclear. However, steric considerations could be among the reasons for this selectivity.

**Scheme 12:**
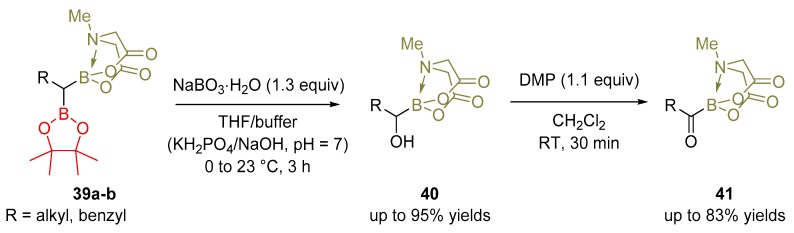
Synthesis of *N*-methyliminodiacetic acid (MIDA) acylboranates via chemoselective oxidation.

Next, Sharma chemoselectively oxidized pinacolate boran functionality over the MIDA boronate of unsymmetrical 1,1-bis(borane) products (**39a**–**b**) to obtain α-hydroxymethyl MIDA boronates (**40**) with good yields (Scheme 12). Thereafter, these α-hydroxymethyl MIDA boronates (**40**) were successfully oxidized into MIDA acylboronates (**41**) using Dess–Martin periodinane (DMP), with moderate to good yields (Scheme 12).

**Scheme 13:**
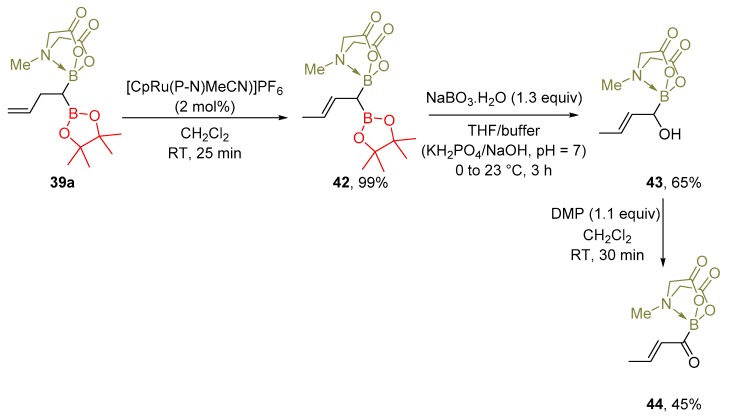
Synthesis of MIDA acylboranates via chemoselective oxidation.

Then, Sharma successfully applied a similar strategy for the synthesis of unique α, β-unsaturated MIDA acyl boronates (**44**) with an acceptable yield (Scheme 13). 

**Scheme 14:**
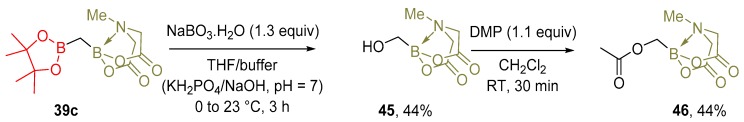
Synthesis of hydroxymethyl MIDA boronates.

Additionally, Sharma applied the same strategy to the unsymmetrical diborylmethane **39c** to obtain hydroxymethyl MIDA **45**. Interestingly, the DMP oxidation of hydroxymethyl MIDA afforded acetoxy MIDA boronate **46** instead of formyl MIDA boronate (Scheme 14) [35].

**Scheme 15:**
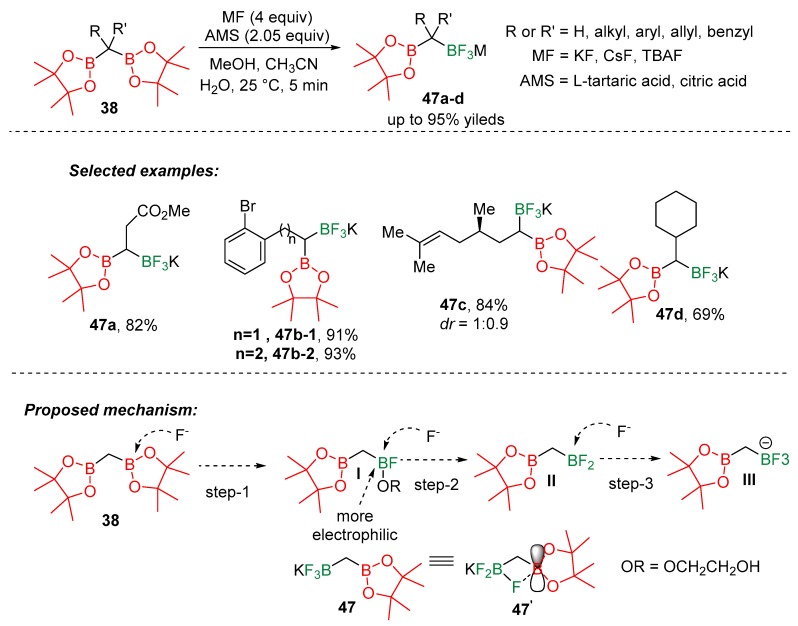
Desymmetrization via nucleophilic trifluorination and the proposed mechanism.

In the same year, the Masarwa research group reported a late-stage desymmetrization of symmetrical 1,1-bis(boryl)alkanes via nucleophilic trifluorination while constructing unsymmetrical 1,1-bis(boryl)alkanes bearing trifluoroborate salts (Scheme 15) [36]. This method was tolerable within a wide range of substrate scopes, with good to excellent yields. Most interestingly, this method did not need any column purification, as the product was obtained upon crystallization. Our group proposed a mechanism to account for this desymmetrization methodology: First, nucleophilic fluoride attacks the vacant *p*-orbital of one of the boron centers in the symmetrical diborane 38 and generates monofluorinated compound I (step 1), which is more electrophilic than the parental symmetrical diborane 38. Hence, it forces the second fluorination (step 2) and then the third fluorination (step 3) at the boron center shown in Scheme 15. Consequently, the newly generated BF_3_ moiety develops a partial negative charge on the fluoride, and the fluoride may stabilize the flanking Bpin group through a fluoride bridge, as shown in 47′, which helps to prevent a subsequent attack by fluoride.

**Scheme 16:**
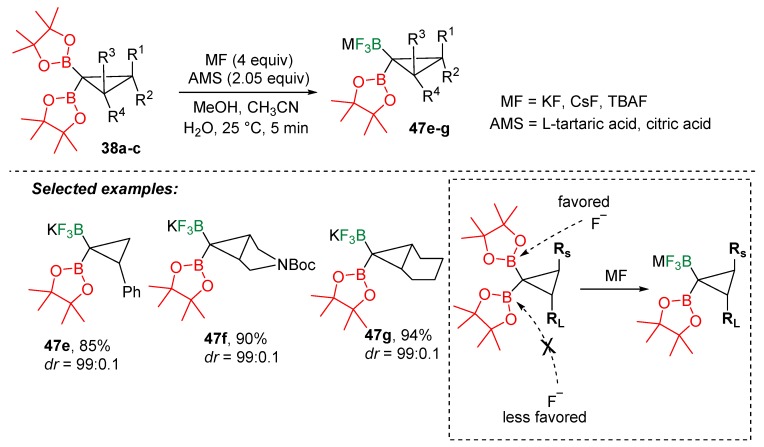
Diastereoselective desymmetrization of cyclopropanes via nucleophilic trifluorination.

Interestingly, this method exhibited excellent diastereo-selectivity when the reaction was performed on the 1,1-bis(boryl)substituted cyclopropanes **38a–c** (Scheme 16). This diastereo-selectivity has been confirmed using 2D-NMR spectroscopic methods. From these results, it can be seen that the diastereo-selectivity mechanism underlying the reaction involves the selective nucleophilic trifluorination of boron, which occupies the less hindered side of cyclopropane.

**Scheme 17:**
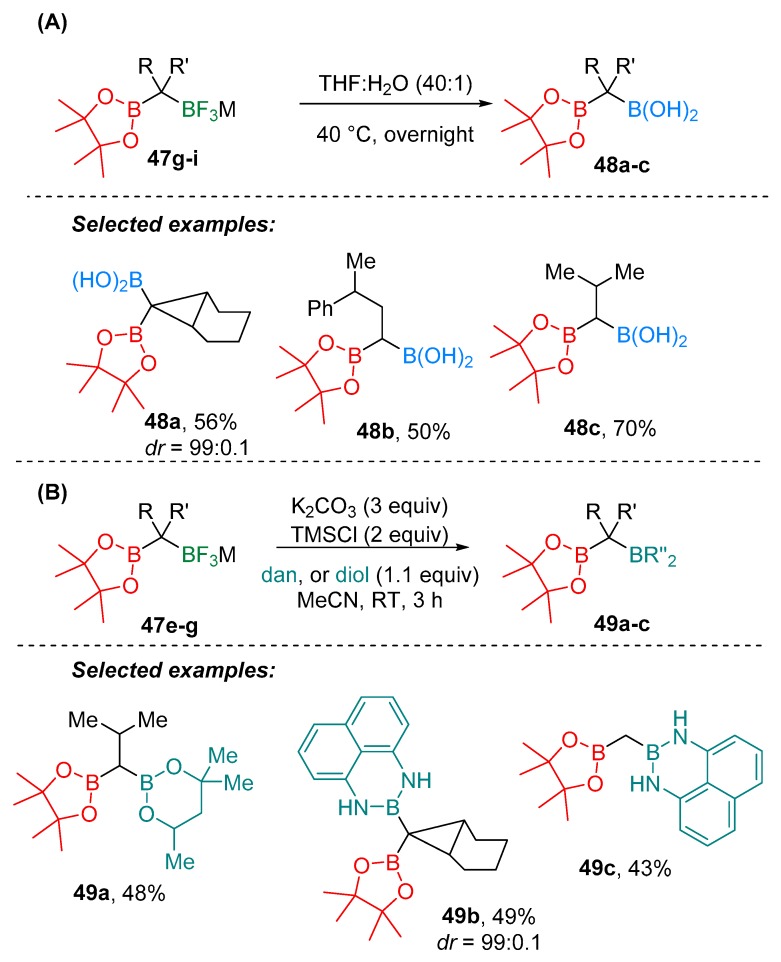
Synthetic transformation of trifluoroborane salt **47** into (**A**) boronic acid **48**; (**B**) unsymmetrical 1,1-bis(borane) product **49**.

These unsymmetrical 1,1-bis(boryl)alkanes, bearing a trifluoroborate group, were utilized for various selective functionalizations. For example, the simple hydrolysis of the trifluoroborate group afforded the 1,1-bis(borane) products **48a–c**, which has a boronic acid moiety (Scheme 17A). Additionally, compound **47** treated with diamines and diols as coupling partners, yielded the unsymmetrical 1,1-bis(borane) products **49a–c** (Scheme 17B).

**Scheme 18:**
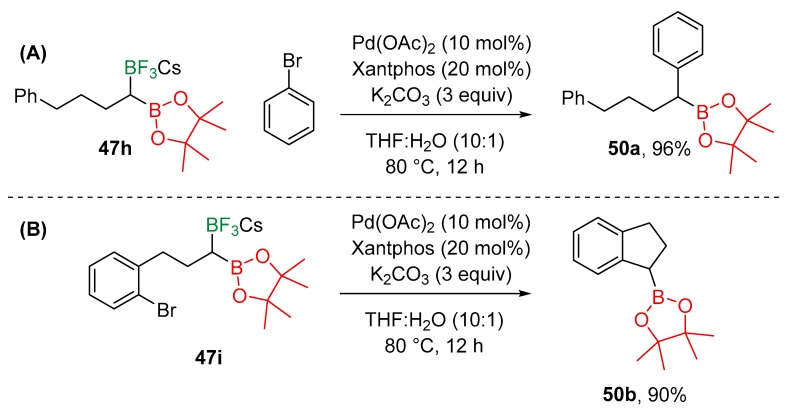
(**A**) Intermolecular Suzuki–Miyaura cross-coupling (SMCC); (**B**) intramolecular SMCC.

Finally, trifluoroborylated unsymmetrical 1,1-bis(boryl)alkanes were also utilized for cross-coupling reactions. Intermolecular palladium(II) catalyzed a cross-coupling reaction with aryl bromide, which afforded arylated product **50a**, which had excellent yield (Scheme 18A). Similarly, an intramolecular cross-coupling reaction of 2-bromo-substituted phenyl substrates under the same reaction conditions yielded the cyclic product **50b**, with a 90% yield (Scheme 18B) [36].

**Scheme 19:**
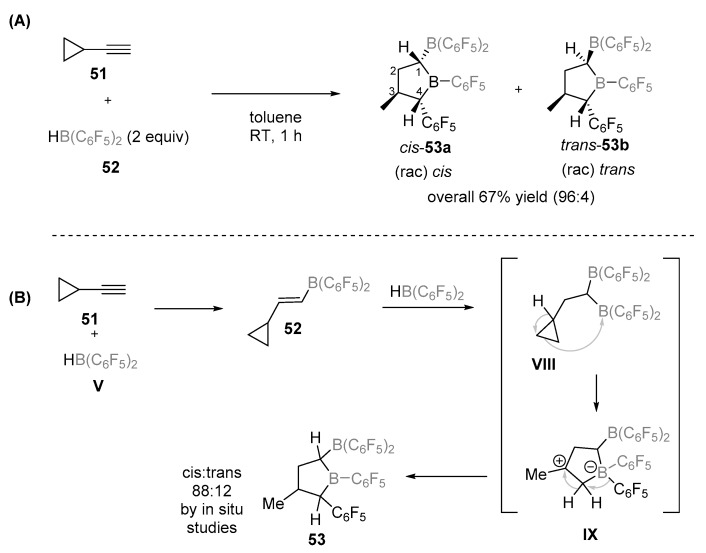
(**A**) Formation and (**B**) rearrangement of tetrahydroboroles.

In 2017, Erker and coworkers reported that Lewis acid induced a cyclopropyl acetylene rearrangement for the synthesis of unsymmetrical 1,1-bis(borane) [37]. In this synthesis, when cyclopropyl acetylene **51** was treated with two equivalents of Piers’s borane **V** [HB(C_6_F_5_)_2_], it resulted in the formation of substituted α-boryl-tetrahydroborole **53a**-cis and **53b**-trans in a 7:1 ratio (Scheme 19A): this was confirmed by in situ NMR spectroscopic studies. Furthermore, they confirmed these structures using X-ray diffraction and found that the pair of substituents at C1 and C4 (in terms of stereochemistry) differed structurally from the *cis* and *trans* isomers.

The mechanism underlying this reaction involves the sequential hydroboration of cyclopropyl acetylene **51** with 2.0 eq. of Piers’s borane **V**, affording 1,1-bis(boryl) compound **VIII**. Then, one of the frustrating borons of product **VIII** acts as a Lewis acid and induces cyclopropyl ring-opening and the hydride sequence. Then, aryl 1,2 shifts to form borole **53** (Scheme 19B). The stereochemical outcome of the reaction largely depends on the least sterically hindered pathway, leading to the major diastereomer *cis*-borole.

**Scheme 20:**
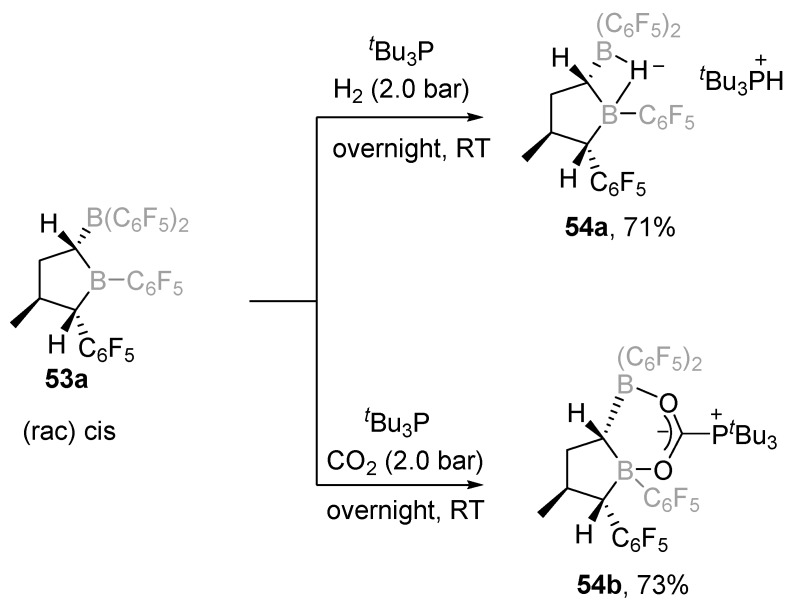
Frustrated Lewis pair (FLP) reaction of unsymmetrical 1,1-bisboroles.

The cyclic 1,1-bis(boryl) contains two Lewis acidic boron sites: they can coordinate only as a frustrated Lewis pair (FLP) or can serve as a dihydrogen splitting reagent. Treatment of a 1:1 ratio of *cis*-borole compound and tri-*tert*-butylphosphine (as a Lewis base with 2.0 bar of hydrogen in pentane solution) resulted in the precipitation of product *cis*-**54a**. Similarly, upon treatment with carbon dioxide instead of hydrogen, a new six-membered cyclic ring was formed, in addition to the borole ring *cis*-**54b** (Scheme 20).

**Scheme 21:**
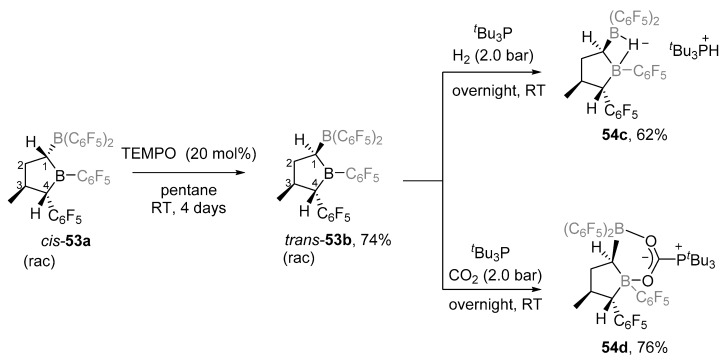
Interconversion of *cis*-borolone into *trans*-borole and its FLP reactions.

Interestingly, Erker and coworkers interconverted *cis*-borole **53a** into *trans*-isomer **53b** by treating it with a catalytic amount of TEMPO (2,2,6,6-tetramethylpiperidine 1-oxyl). This reaction followed reversible H-abstraction at the activated C_1_ position of the heterocycle. After they subjected *trans*-borole to a similar FLP reaction, they obtained products **54c** and **54d** in good yields (Scheme 21) [37].

**Scheme 22:**
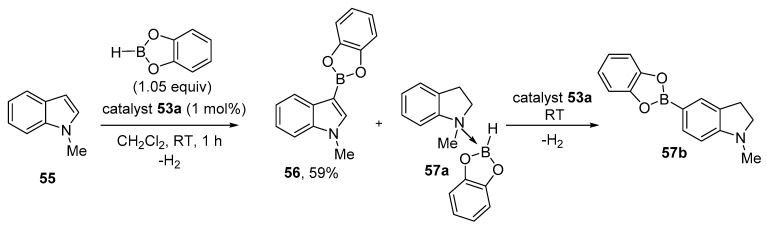
1,1-bis(borane)-catalyzed borylation of *N*-methylindole.

In the same year, Erker and coworkers reported that cyclic 1,1-bis(borane) **53** catalyzed the hydroboration of *N*-methylindole. Here, 1,1-bis(borane) compounds were utilized as effective catalysts for C–H bond activating the borylation of *N*-methylindole with catechol borane. This reaction afforded 3-boryl-*N*-methylindole with a 59% yield and *N*-methylindoline with a Lewis pair adduct (with HBcat). Furthermore, this adduct, upon treatment with the same catalyst for several hours, yielded a 5-boryl-*N*-methylindoline product through the evolution of molecular hydrogen (Scheme 22) [38].

## 3. Unsymmetrical sp^2^-Centered 1,1-bis(boron) Compounds: Synthesis and Applications

**Scheme 23:**
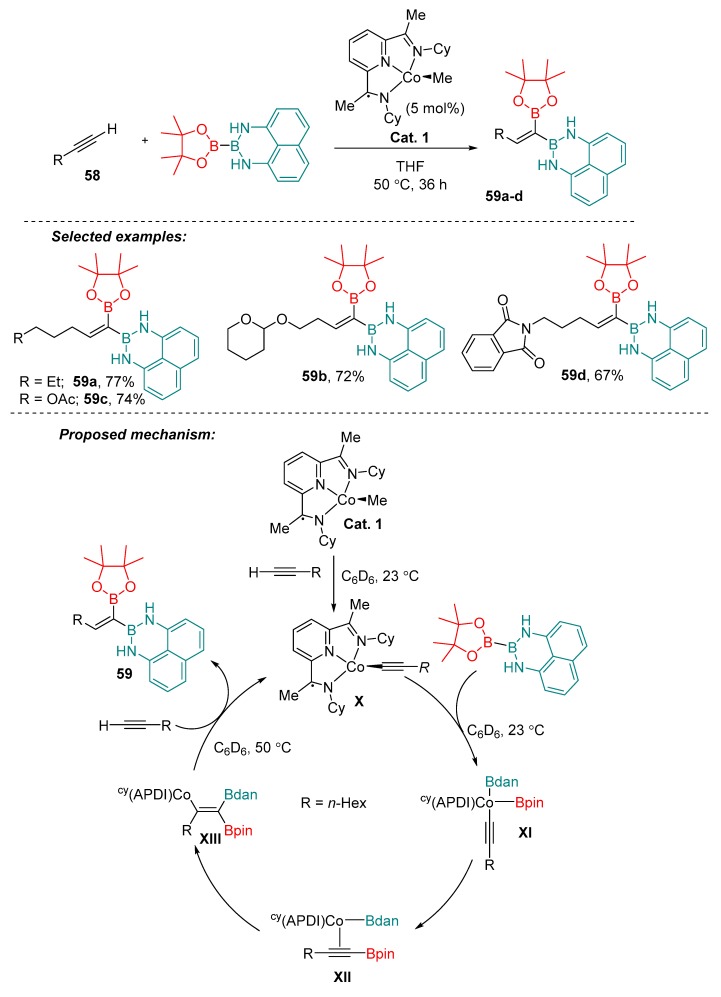
Cobalt-catalyzed stereoselective 1,1-diboration of terminal alkynes with pinB–Bdan.

In 2007, Chirik and coworkers developed a cobalt that catalyzed the 1,1-diboration of readily available terminal alkyne **58** with an unsymmetrical (pinB–Bdan) diboron reagent for the synthesis of stereoselective trisubstituted 1,1-bis(boryl)alkenes (**59a–d**), with good yields (Scheme 23) [39].

The mechanism proposed by Chirik’s group involved the initial formation of cobalt acetylide (**X**), which, upon reacting with pinacolborane, yielded compound **XI**, which had more Lewis acidic boron substituent (Bpin). This could transfer to the alkyne, and the resulting alkynyl−BPin cobalt complex (**XII**) underwent syn-borylcobaltation, selectively affording **XIII**, which finally produced the stereoselective alkene **59**.

**Scheme 24:**
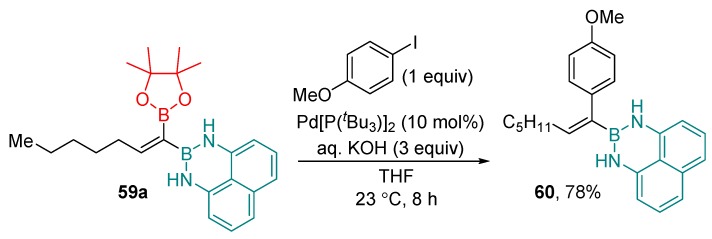
Selective Suzuki–Miyaura cross-coupling at Bpin.

Taking advantage of the different chemical reactivities of two boron moieties (Bpin, Bdan) in 1,1-unsymmetrical bis(boryl)alkenes (**59**), an SMCC reaction was carried out with aryl iodides to afford corresponding (*Z*)-alkenes (**60**), which had an extended conjugation and good yields. Interestingly, they observed that the cross-coupling took place selectively at the Bpin moiety over Bdan (Scheme 24) [39]. The whole methodology, which includes the 1,1-diboration of alkynes (Scheme 23) and the cross-coupling reaction of 1,1-unsymmetrical bis(boryl)alkenes (Scheme 24), represents a formal 1,1-carboboration of hept-1-yne with Ar–Bdan [40,41,42].

**Scheme 25:**
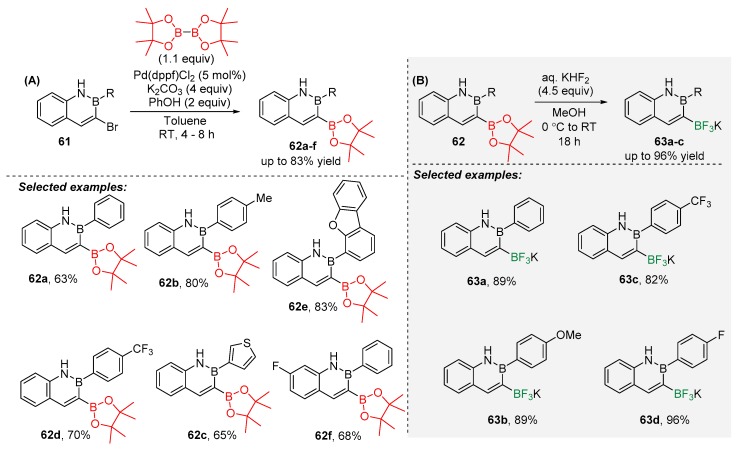
(**A**) Pd-catalyzed borylation of brominated 2,1-borazaronaphthalenes; (**B**) synthesis of 3-BF_3_K-2,1-borazaronaphthalenes.

In 2018, the Molander group reported the borylation of 3-bromo-2,1-borazaronaphthalenes (**61**) with boronic acid pinacol esters, affording 3-boryl-2,1-borazaronaphthalene **62a–f** (1,1-unsymmetrical bis(boryl)alkenes) [43]. These borazaronaphthalenes (**62**) also exhibited an umpolung character in cross-coupling reactions. This method allows for the synthesis of a wide range of heterocycles with different substituents at the boron center, with electron-rich and electron-poor aryl and heteroaryl groups and up to an 83% yield (Scheme 25A). Next, compound **62** was converted into organotrifluroborate salt (**63**) by treating it with commercially available KHF_2_ as a fluoride ion source (Scheme 25B).

**Scheme 26:**
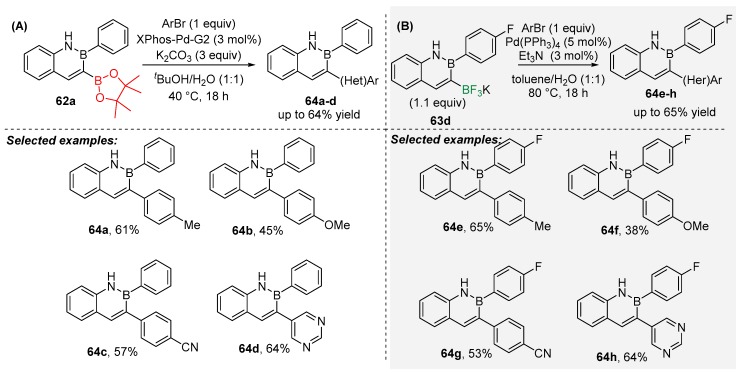
Scope of the direct cross-coupling with (**A**) 3-Bpin-*B*-phenyl-2,1-borazaronaphthalene and (**B**) 3-BF_3_K-*B*-phenyl-2,1-borazaronaphthalene.

Later, they also utilized the bis-boryl compounds **62a** and **63d** for a palladium-catalyzed cross-coupling strategy with a variety of aryl halides containing an electron-withdrawing group or an electron-donating group, which yielded the corresponding coupling products **64a–h** (Scheme 26) [43].

**Scheme 27:**
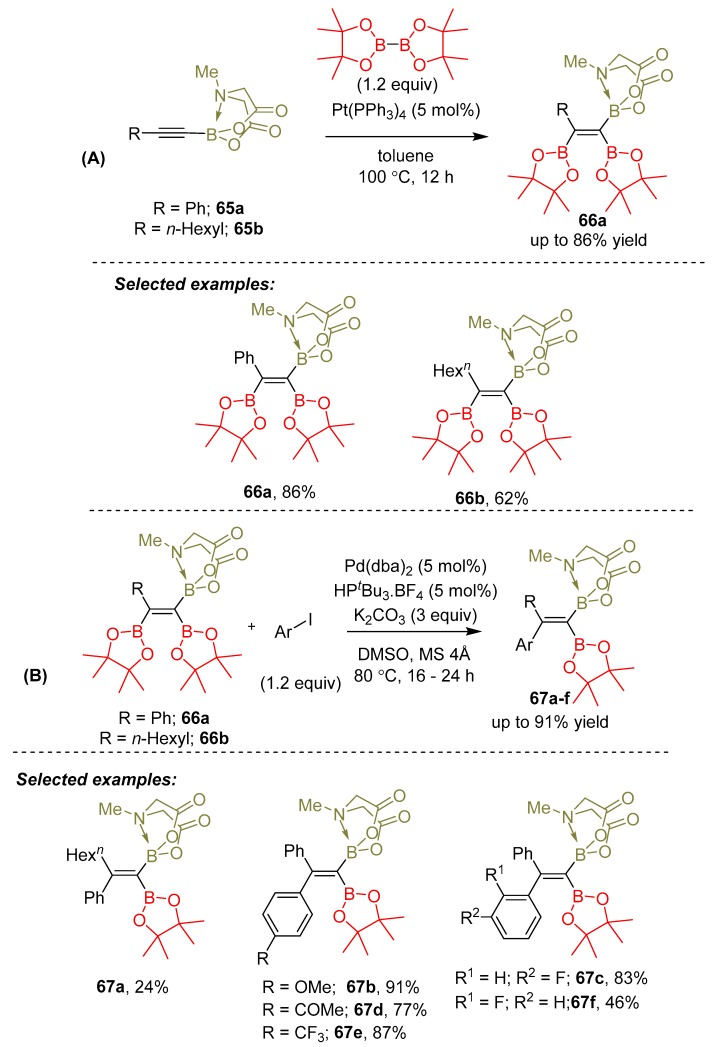
(**A**) Stereoselective platinum-catalyzed 1,2-diborylation of alkyne; (**B**) chemoselective Suzuki–Miyaura coupling of **66**.

In 2014, the Nishihara research group reported the platinum-catalyzed diborylation of 1-phenylethynyl MIDA boronate **65a** with bis(pinacolato)diboron, affording stereoselective 1,1,2-triboryl-2-phenylethene **66a** with an 86% yield [44]. Under similar reaction conditions, they also extended diboration with the aliphatic 1-alkynyl MIDA boronate **65b**, yielding the 1,1,2-triboryl-2-hexylethene **66b**, as shown in Scheme 27A. Furthermore, 1,1,2-triboryl-2-phenylethene, **66**, was successfully applied to chemoselective palladium-catalyzed Suzuki−Miyaura coupling with aryl halides bearing electron-donating and electron-withdrawing groups. Under optimized reaction conditions, they synthesized a library of synthetically useful 1,1-bis(boryl)olefins, **67a–f**, with up to 91% yields (Scheme 27B).

**Scheme 28:**
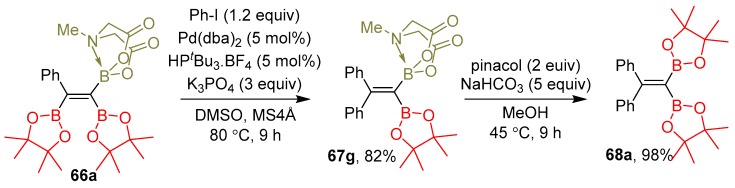
Determination of the stereochemistry of arylated product **67**.

To determine the *(Z)*-configuration of the chemoselective arylated product **67**, they carried out Suzuki−Miyaura coupling of 1,1,2-triboryl-2-phenylethene **66a** and iodobenzene to afford the arylated unsymmetrical 1,1-bis(boryl)-2,2-diphenylethene **67g**, with 82% yield. Then, transformation of the BMIDA group into Bpin afforded the symmetrical 1,1-bis(boryl)-2,2-diphenylethene **68a** at a 98% yield, which matched with earlier reported spectroscopic data. This experimental result clearly suggests that selective cross-coupling takes place at the Bpin group, which is geminal to the aryl moiety (Scheme 28).

**Scheme 29:**
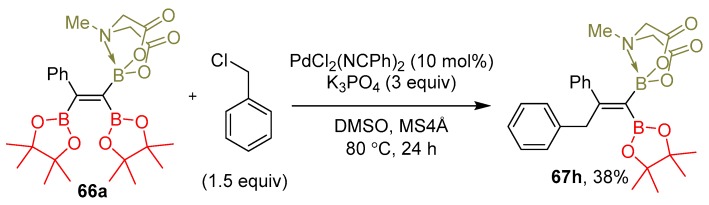
Suzuki–Miyaura coupling of **66a** with benzyl chloride.

Furthermore, they changed the electrophile ArI into BnCl (1.5 equiv) for an SMCC reaction with compound **66a** and observed only one isomer of unsymmetrical 1,1-bis(boryl) alkene **67h**, with a moderate yield (Scheme 29) [44].

**Scheme 30:**
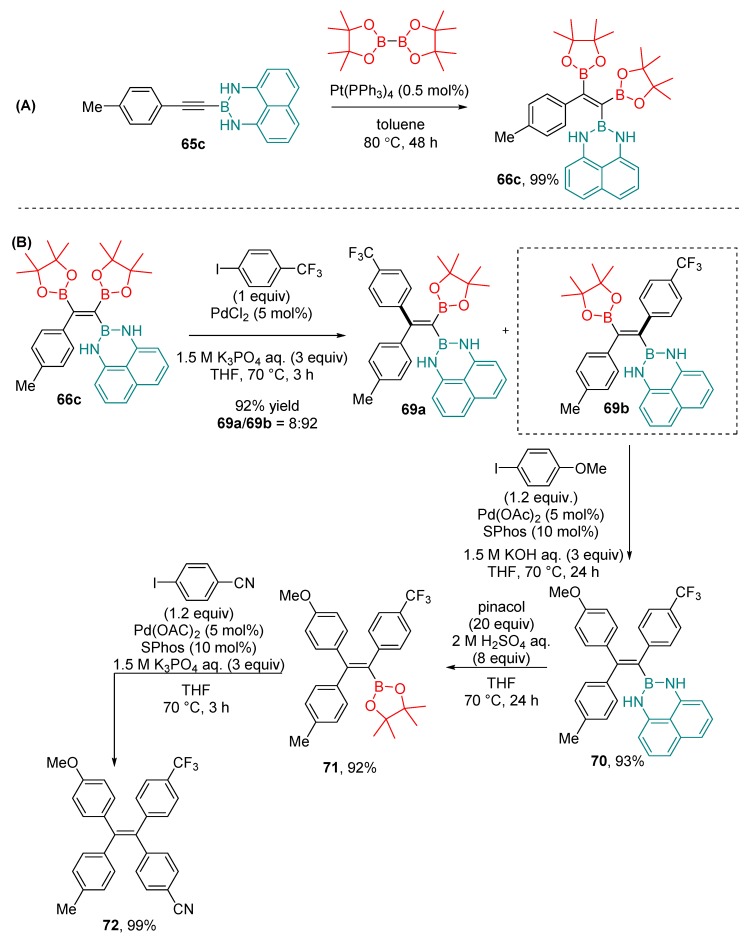
(**A**) Platinum-catalyzed 1,2-diboration of alkyne with B_2_(pin)_2_; (**B**) Suzuki–Miyaura cross-coupling reactions to afford the tetra-arylalkene **72**.

In 2019, the Tsuchimoto group reported that the Pt-catalyzed diboration of alkyne had terminal Bdan with pinB–Bpin, resulting in the 1,1,2-triboryalkene **66c**, which had perfect stereoselectivity without suffering direct activation by the platinum complex [45]. Compared to other reports on the diboration of alkynes [46,47] with pinB–Bpin, this method afforded an excellent yield (Scheme 30A).

In 2019, utilizing the *tetra*substituted triborylalkene **66c**, for the first time the Tsuchimoto group successfully synthesized *tetra*substituted aryl alkenes (**72**) through sequential SMCC reactions. In the triborylalkene **66c**, the C*sp^2^*-B bond is inactive; thus, it is very unreactive under SMCC conditions. Of the remaining two C*sp^2^*-B bonds, one is at the transposition of the aryl group, which can possibly increase the nucleophilicity of the boron atom through electron flow: it reacts more facilely than does the other under Pd(II) SMCC conditions. In the first SMCC reaction, 4-iodobenzotrifluoride was treated under ligand-free conditions, affording **69** in very good yield (with regioselectivity). In the second SMCC arylation, 4-iodoanisole reacted preferentially with C*sp^2^*-Bpin over C*sp^2^*-Bdan, affording **70**. Since Bdan is unreactive toward SMCC, it was converted into Bpin under acidic conditions with pinacol. In the final sequence of SMCC arylation, 4-iodobenzonitrile was treated with C*sp^2^*-Bpin to produce the tetra-arylalkene **72**. Importantly, during all of the SMCC reactions observed, there was only one stereoisomer with a high yield (Scheme 30B) [47].

**Scheme 31:**
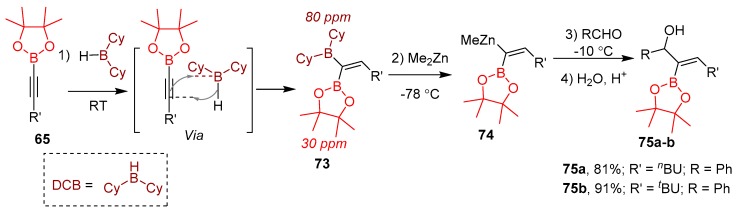
Synthesis of allylic alcohols from alkyne via unsymmetrical diborylalkenes.

In 2008, the Walsh group described the reaction of stable pinB-substituted alkynes (**65**) with dicyclohexyl borane to afford unsymmetrical 1,1-bis(boryl)alkene species (**73**) [48]. Compound **73** was not isolated and exhibited two peaks at 30 ppm and 80 ppm in a crude ^11^B-NMR spectrum. In addition, crude ^1^H-NMR of **73** exhibited only one isomer in the hydroboration reaction. Later on, they successfully utilized a 1,1-bis(boryl)alkane species for chemoselective transmetallation with an organozinc reagent (in place of Cy_2_B) to afford the boron/zinc heterobimetallic reagent **74**; they then added it to an aldehyde to obtain pinB-substituted *(E)*-allylic alcohols (**75**) with good yields. By utilizing this method, they synthesized a library of secondary alcohols via hydroboration and transmetallation, followed by aldehyde treatment with pinB-substituted alkynes (**65**) (Scheme 31) [48].

**Scheme 32:**
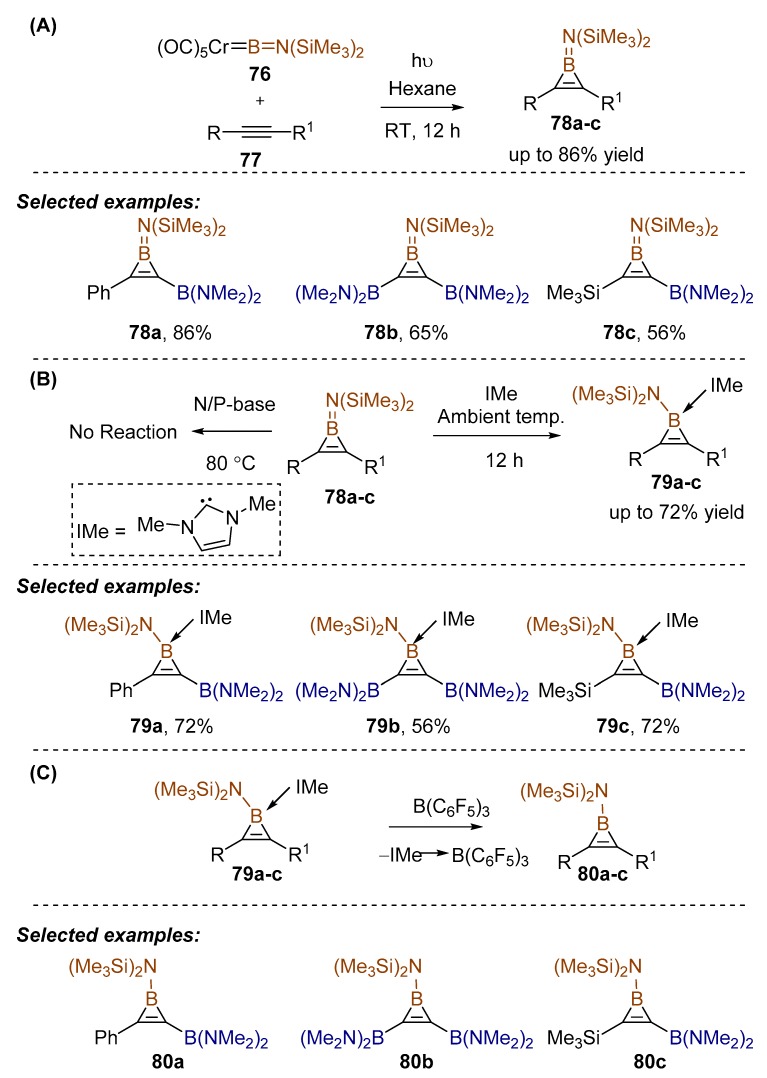
(**A**) Photolytic synthesis of borirenes; (**B**) reactivity of borirenes toward bases; (**C**) dequarternization of borirenes.

In 2013, the Braunschweig group demonstrated a photolytic approach to creating a new class of three-membered borirenes using an aminoboryl complex [(OC)_5_Cr=B=N(SiMe_3_)_2_] in the presence of a series of mono- or bis(boryl) alkynes: 1-phenyl-2-bis-(dimethylamino)borylethyne, bis{bis-(dimethylamino)boryl}ethyne, and 1-trimethylsilyl-2-bis-(dimethylamino)borylethyne [49]. As depicted in Scheme 32A, aminoboryl complex **76** was irradiated with alkyne **77** at room temperature to produce the desired unsymmetrical 1,1-bis(boryl) alkenes (i.e., iminoboranes) (**78a–c**) (Scheme 32A).

Generally, aminoboranes (i.e., compound **80**) do not follow quaternization, since the *p*-orbital of boron is filled by the π-basic amino group. The utility of iminoboranes (**78**) was established by the synthesis of quaternary aminoboranes (**79**): these kinds of aminoboranes are very rare, according to the literature (Scheme 32B). The iminoborane **78** did not react with DMAP (4-(dimethylamino)pyridine), pyridine, PCy_3_, or PMe_3_ even at 80 °C, whereas it reacted with IMe (IMe = 1,3-dimethyl-2,3-dihydro-1*H*-imidazole) at ambient temperature to produce quaternization in the endocyclic boryl group. Interestingly, the Braunschweig group observed that there was no quaternization of the exocyclic boryl groups even upon the addition of an excess of IMe (Scheme 32B).

The dequarternization of borirenes also afforded the parental unsymmetrical 1,1-bis(boryl)alkanes and alkenes (**80**) in the presence of B(C_6_F_5_)_3_ (Scheme 32C) [49].

**Scheme 33:**
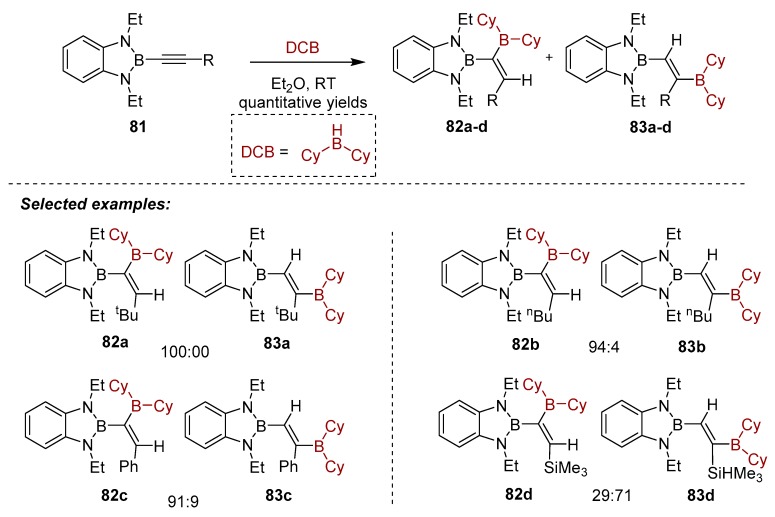
Hydroboration of alkynes with HBCy_2_ (dicyclohexylborane).

In 2013, Weber et al. reported the hydroboration of 2-alkyl- and 2-aryl-ethynyl-1,3,2-benzodiazaboroles with dicyclohexylborane (DCB) under metal-free conditions at room temperature, affording the *cis*-1,1-bis(boryl)alkene **82** as a major regioisomer, with quantitative yields [50]. Interestingly, they also observed the hydroboration of 2-silylethynyl-1,3,2-benzodiazaborole with DCB, which afforded 1,1-bis(boryl)alkene (**82**) as a minor regioisomer and *trans*-1,2-bis(boryl)alkene as a major regioisomer (**83**), with quantitative yields (Scheme 33) [50].

**Scheme 34:**
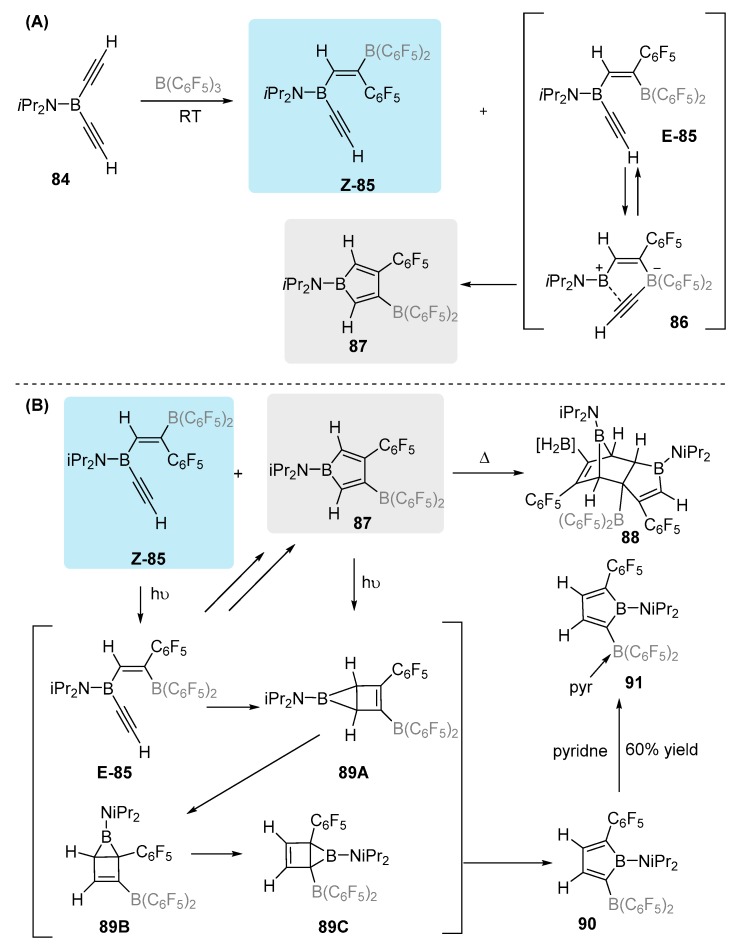
(**A**) 1,1-carboboration of **84**; (**B**) the synthesis of 1,1-bis(borane) from a mixture of 1,3,4-trisubtituted boroles and **Z-7**.

In 2015, the Erker group reported the preparation of 1,2,5-trisubstituted boroles containing 1,1-bis(boryl) groups from the bis(ethynyl)borane **84** and B(C_6_F_5_)_3_ via a 1,1-carboboration sequence followed by di-π-borane rearrangement [51]. Initially, the reaction of bis(ethynyl)borane with B(C_6_F_5_)_3_ resulted in 44:56 mixtures of the 1,1-carboboration product ***Z*-85** and the trisubstituted borole **87** via **86**, which was isolated after treatment with pyridine (Scheme 34A).

Under thermal conditions, a mixture of ***Z*-85** and **87** afforded the [4 + 2] cycloaddition product **88**, which was formed after the dimerization of reactive borole **87**. The Erker group observed that the unreacted ***Z*-85** remained under thermal conditions (Scheme 34B). Since compound ***Z*-85** was not isomerized thermally, they tried photolysis. Under photolysis, ***Z*-85** isomerized to ***E*-85**, which can easily be converted into **87**, as shown in Scheme 34A, and compound **87** also cleanly isomerized to the new trisubstituted borole **90** via di-π-borane rearrangements (two formal 1,3-boron migrations) followed by ring opening. Consequently, the photolysis of a solution of the ***Z*-85** + **87** mixture resulted in 1,1-bis(boryl)alkanes and alkenes (**90**) (1,2,5-trisubstituted boroles) in quantitative conversion, which was isolated after treatment with pyridine (Scheme 34B) [51].

**Scheme 35:**
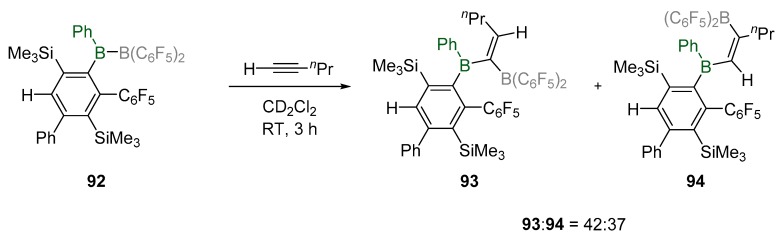
The diboration of alkyne.

In 2018, the same group prepared a 1,1-bis(boryl)alkene by reacting tetraaryldiborane(4) **92** with 1-pentyne (Scheme 35) [52].

Under thermal conditions, diborane(4) underwent 1,2-migration in its acetylenic hydrogen along the alkyne π-bond to afford 1,1-bis(boryl)alkene **93**, along with **94**, in a 42:37 ratio. During the reaction, one B–B bond was cleaved, and new C–B bonds were formed (Scheme 35) [52].

## 4. Conclusions

In this review, we wished to emphasize the importance of unsymmetrical 1,1-bis(boryl) species as unique building blocks that lead to synthetically new connections. For their preparation, we mainly focused on a few main strategies: 1) the metal-catalyzed conjugate borylation of β-boronylacrylates, the hydroboration of boryl-alkenes, the 1,1-diboration of alkynes, the 1,2-diboration of borylated alkyne, and the borylation of brominated 2,1-borazaronaphthalenes; 2) the metal-free insertion of diazo-compounds into pinB–Bdan, the hydroboration of borylated alkenes, the hydroboration of borylated alkyne, and Piers’s borane (which induces cyclopropyl rearrangement and the diboration of alkynes); 3) the late-stage desymmetrization of symmetrical 1,1-bis(boryl)alkanes; and 4) the photolytic synthesis of diboranes. Additionally, transformations of unsymmetrical 1,1-bis(boryl) species were extensively studied in this review. Unsymmetrical 1,1-bis(boryl) species are unique because two boryl groups exhibit different chemical reactivities toward chemoselective SMCC reactions, including sequential cross-coupling reactions with alkyl/aryl halides, chemoselective oxidation, hydrolysis, and transmetallation. We also revealed the rare catalytic activity of 1,1-bis(borane). Due to its potential in the synthesis of unsymmetrical 1,1-bis(boryl)alkenes, this field will continue to grow rapidly, and more appealing transformations are expected to appear in the years to come. 

Moreover, these classes of unsymmetrical 1,1-bis(boryl) compounds have the potential to be linked to important materials in the late stages of their synthesis, which then (by applying a variety of selective reaction conditions to unsymmetrical 1,1-bis(boryl) units) leads to multiple different functional groups in order to create new chemical libraries of bioactive compounds, drugs, and natural products that are demonstrative of a large serving of these compounds. Therefore, unsymmetrical 1,1-bis(boryl) and its transformations hold great promise in contributing to diversity-oriented synthesis.

Future approaches to expand the synthetic utility of these unsymmetrical 1,1-bis(boryl) compounds (**III** and **IV**) can be carried out by selectively transforming their C–B bonds into C–heteroatom bonds.
